# Macrofollicular variant of papillary thyroid carcinoma with metastasis to femur

**DOI:** 10.1186/s13044-020-00083-w

**Published:** 2020-06-13

**Authors:** Fernando Candanedo-Gonzalez, Diana Rodriguez-Orihuela, Julian Arista-Nasr

**Affiliations:** Department of pathology, Instituto Nacional de Ciencias Medicas y Nutricion Salvador Zubiran, Av. Vasco de Quiroga No. 15. Col: Belisario Dominguez Seccion XVI, Delegacion: Tlalpan, 14080 Ciudad de Mexico, Mexico

**Keywords:** Thyroid carcinoma, Macrofollicular variant of papillary thyroid carcinoma, Bone metastases, Femur

## Abstract

**Background:**

Macrofollicular variant of papillary thyroid carcinoma (MFV-PTC) is a rare subtype with histological features and an indolent clinical course that can be confused with nodular goiter or follicular adenoma. However, on rare occasions it may show more aggressive biological behavior. Only two cases of MFV-PTC with bone metastases have been reported previously. We describe the clinical-pathological characteristics of a man with MFV-PTC that developed femur metastasis. This the first case of MFV-PTC with femur metastases diagnosed histologically by means of an image-guided core needle biopsy in English literature.

**Case presentation:**

A 33-year-old man presented two years earlier with swelling in the right neck associated with weight loss and pain in the left knee. Neck ultrasonography showed hyperechogenic and hypogenic nodular images in the thyroid’s right lobe. A fine needle aspiration biopsy specimen was initially interpreted as nodular hyperplasia. A CT showed a large tumor involving right thyroid lobe with trachea and vascular structures displaced to the right, and a total thyroidectomy was performed. Subsequently, a MRI of the knee confirmed the presence of a destructive tumor in the femur. A guided core-needle biopsy of the knee tumor showed the presence of metastatic MFV-PTC. Re-assessment of the histology from thyroidectomy was also consistent with MFV-PTC. A whole-body ^18^F-FDG PET/CT showed presence of lesions in the left anterior costal arch, metaphysis of the left femur and in the sternum handle. Therefore, he received 200 mCi radioactive iodine, and 40 Gy of radiotherapy to left costal arches and knee, which decreased his symptoms. Currently, after 10 months of follow-up, the patient is alive with bone tumor activity.

**Conclusions:**

Our case supports the view that, on rare ocassions, MFV-PTC may show a more aggressive biological behavior than expected. The synchronous or asynchronous presence of one or more bone lesions, should raise the suspicion of metastasis. Given the suspicion, it is necessary to take a biopsy to confirm histologically. Only a careful analysis of the architectural and cytological characteristics of goiter or hyperplastic nodules will allow to recognize this rare variety of carcinoma.

## Background

Macrofollicular variant of papillary thyroid carcinoma (MFV-PTC) was described in 1991 by Albores-Saavedra et al [1]. It is a rare variant of papillary carcinoma with a frequency of 2.6% [2], characterized by highly dilated thyroid follicles in more than 50% of the neoplasm and resembles thyroid adenoma or nodular colloid goiter. Most have a good prognosis and are generally encapsulated. In the original series of 17 patients, two presented with lymph node metastases and later, other authors found similar cases [3–5]. In 2009, Cardenas et al [6] reported two patients who presented with bone metastases. We describe the clinical pathological characteristics of a 33-year-old man with MFV-PTC that was initially misdiagnosed as a benign goiter following a fine-needle aspiration (FNA) biopsy of the thyroid, with synchronous metastases to the left femur and ribs. This is the first report of MFV-PTC with femur metastases histologically documented by means of an image-guided core needle biopsy in English literature.

## Case presentation

**Fig. 1:**
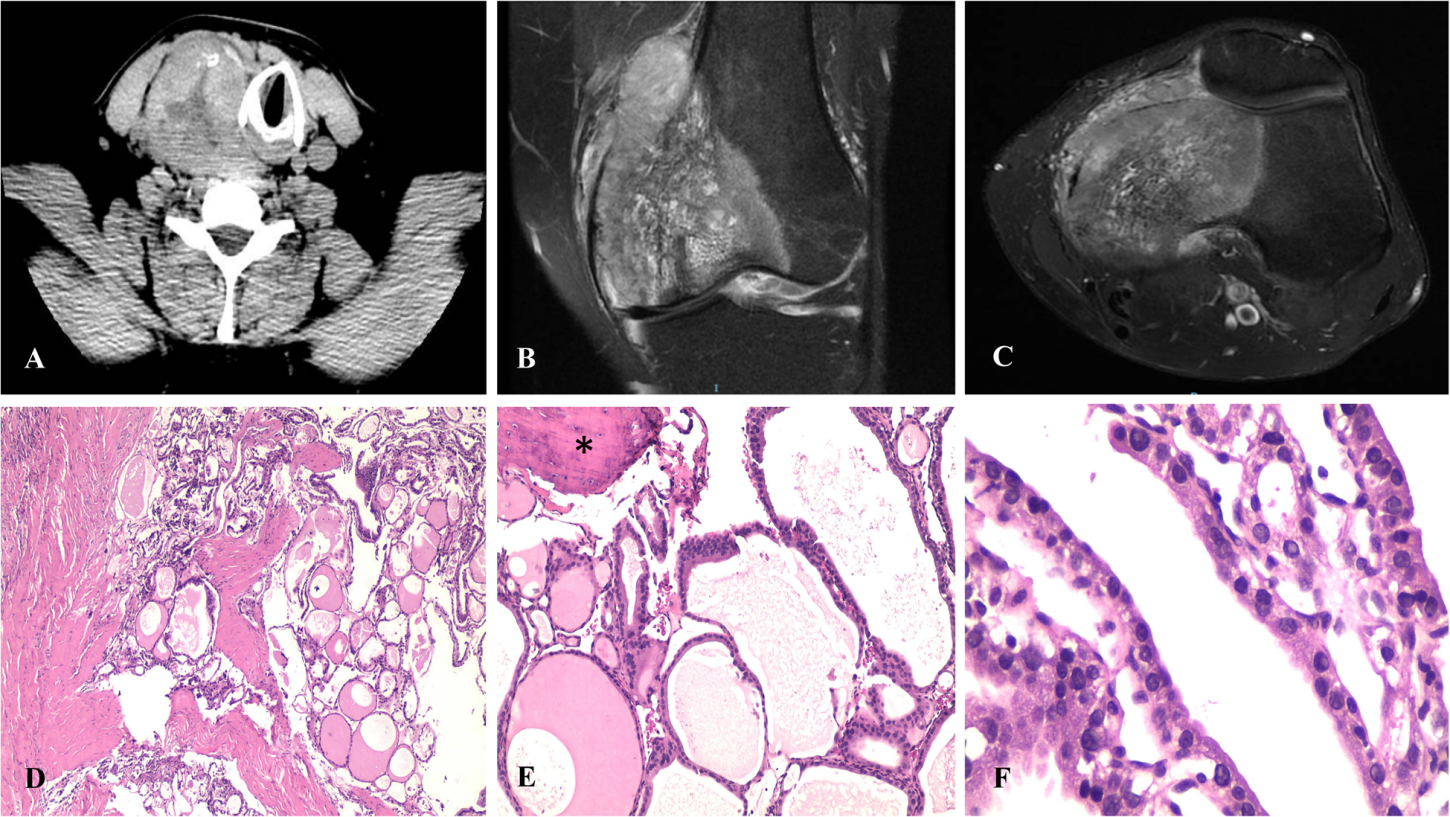
Metastatic macrofollicular thyroid carcinoma to femur. **a** CT showed the presence of a tumor involving right thyroid lobe with central necrosis that displaced the upper third of the trachea and the vascular structures on the right side of the neck; **b** and **c** MRI showed a destructive neoplasm in the diaphysis and epiphysis of the femur; **d** The neoplasm showed large follicles in more than 50% of the neoplastic follicles; **e** Immersed in the metastasis of macrofollicular variant papillary thryroid carcinoma, a bone spicule was observed (*); **f** Follicles were lined by atypical nuclei and some showed pseudoinclusions

A 33-year-old mestizo man, who in 2017, noticed a swelling in the right neck associated with weight loss and pain in the left knee; hence he consulted a doctor outside of our institution. On physical examination, he had an asymmetrical neck, with trachea deviated to the left, and a hard mass, in the thyroid gland, not fixed to deep planes. No adenomegalies were palpable. A year later he presented dysphagia to solids, asthenia, adinamia, dyspnea and snoring. Thyroid ultrasound showed hyperechogenic and hypoechogenic nodular lesions with necrotic center and microcalcifications in the right lobe. The nodules measured between 10 and 18 mm in diameter with increased perfusion around the nodular lesions. The right lobe measured 95x52x67 mm and the left lobe measured 39x16x17 mm. Angio-Doppler showed diffuse isoechoic feature and was classified as TI-RADS 4A (Thyroid Imaging and Reporting System). No locoregional lymph node growth was observed. A FNA biopsy of the thyroid gland was initially interpreted as nodular hyperplasia (Bethesda 2). Therefore, he was referred to our institution for further management. Laboratory investigations showed serum thyroglobulin 1359.67 ng/mL (0–36.8), thyroid stimulating hormone (TSH) 3.57 mlU/L (0.3–5), total tetraiodothyronine (T4T) 8.45 μg/dL (5.91–12.56), free tetraiodothyronine (FT4) 0.74 ng/dL (0.63–1.34), total triiodothyronine (T3T) 1.91 ng/mL (0.64–1.81), free triiodothyronine (FT3) 4.21 pg/mL (2.5–3.9), T3 resin uptake 42.8% (24–37), anti-thyroid peroxidase antibodies < 1:1200 IU/mL (negative). In April 2019, a computed tomography (CT) of the neck was performed that confirmed the presence of a tumor involving the right thyroid lobe with central necrosis that displaced the upper third of the trachea and vascular structures on the right side of the neck with high level of peripheral reinforcement in the sequence with contrast medium (Fig. 1a). In August 2019, total thyroidectomy was performed. On the other hand, the examination of the left knee showed a swelling in the medial side, which was painful to mobilize and had a hard consistency. A magnetic resonance imaging (MRI) of the knee confirmed the presence of a destructive tumor in the diaphysis and epiphysis of the femur, which measured 82x54x52 mm, involving the articular medial intercondylar component and the posterior cruciate ligament (Fig. 1b and c). An imaging guided core-needle biopsy of the knee tumor showed a presence of metastatic thyroid carcinoma. In October 2019, a whole-body ^18^F-fluorodeoxyglucose positron emission tomography and computed tomography (^18^F-FDG PET/CT) was performed, which showed the presence of a 5.3 × 4.1 cm lesions in the left anterior costal arch with 4.6 SubMax. The scan also showed a 6.9 × 4.7 cm lesion with SubMax of 7.1 in the left femur and a mixed lesion in the sternum handle, with SubMax of 2.5. Therefore, in November, 2019 he received a therapeutic dose of radioactive iodine (RAI) of 200 mCi and zoledronic acid, as well as 40 Gy (divided in 10 fractions) of local external radiotherapy to the left costal arches and the knee, which decreased his symptoms. Currently, after 10 months of follow-up, the patient is alive with bone tumor activity.

## Materials and methods

The tissue was fixed in 10% buffered formaldehyde and paraffin embedded. For immunohistochemistry (IHC) analysis, 5 μm sections of a representative block were obtained. The following antibodies were used: Thyroid Transcription Factor-1 (TTF-1) and thyroglobulin. IHC analyses were performed on an automated immunostainer (Ventana, Biotek System, Tucson, Arizona) with appropriate positive and negative control run concurrently. Briefly, paraffin sections were mounted on charged glass slides, air-dried over-night, and then deparaffinized. To enhance the immunostaining, a heat-induced epitope-retrieval procedure was performed. After incubation with blocking serum, sections were incubated with primary antibodies, followed by a biotinylated polyvalent secondary antibody solution. Sections were then incubated with horseradish peroxidase conjugated avidin-biotin complex, followed by 3, 3-diaminobenzidine and hydrogen peroxidase.

### Pathologic findings

Macroscopically, the right thyroid lobe weighed 130 g and measured 8.6 × 7.5 cm. At the cut, multiple nodules of variable size were identified with a central zone with multiple calcifications. The left thyroid lobe and isthmus weighed 8.5 g, and measured 4.5 × 2.0 cm.

Microscopically, both the right thyroid lobe and the tumor in the femur showed a neoplasm composed mainly of macrofollicles with abundant colloids, and a complete absence of well-formed papillary structures (Fig. 1d). The nuclei of the neoplasic cells showed ground-glass opacity with nuclear overlapping (Fig. 1e and f). No mitosis or necrosis were observed. On IHC, the neoplasm cells were positive for thyroglobulin and TTF-1 diffusely.

## Discussion

Papillary thyroid carcinoma (PTC) is the most common malignant neoplasm of the thyroid gland. It represents 80% of all thyroid malignancies [7]. PTC has a morphologically broad spectrum. The MFV-PTC is a rare subtype, which was defined for the first time by Albores-Saavedra et al., in 1991 [1]. In the original description it was established that the follicles of MFV-PTC occupied over 50% of the cross-sectional areas of the tumors. The macrofollicles were lined either by cells with enlarge ground-glass clear nuclei, and nuclear grooves or cuboidal cells with hyperchromatic nuclei [1]. All patients were females ranging in age from 15 to 69 years (mean, 35.4 years). MFV-PTC is a well-differentiated neoplasms usually with an indolent clinical course. Despite high survival rates, some patients may present with local recurrence, and metastasis and this may require more aggressive treatment.

The most frequent sites of metastasis in PTC are locoregional lymph nodes, especially cervical and mediastinal nodes. On the other hand, the most common sites of distant metastases are lungs and bones. Bone metastases most often occur in the scapula, sternum and ilium. However, bone metastases are most often caused by follicular thyroid carcinoma, while only 1.4 to 7% of PTCs produce bone metastases [8–10]. There are retrospective studies that have suggested that the MFV-PTC has a better prognosis as compared to the classical form, with its lower rate of lymph node metastases [11]. Despite being a low grade malignant neoplasm, cases with nodal and pulmonary metastases from MFV-PTC have been reported. However, there are only two cases of particularly aggressive MFV-PTC with extensive bone dissemination, previously reported by Cardenas et al [6]. The first case corresponded to a 59-year-old woman with a metastasis in the occipital bone, ribs, thoracic, lumbar spine and pelvic bones. Thyroidectomy showed a 3 cm diameter MFV-PTC. The second case was an 81-year-old man with a thyroid nodule that was originally interpreted as a nodular colloid goiter. Three years later, he developed a neoplastic mass in his right shoulder that was confirmed to be MFV-PTC. Following the review of the thyroid nodule, the lesion was reclassified as MFV-PTC. Subsequently, other bone and lung lesions were documented. The evolution was unfavorable and he died. We report the third case of bone metastasis from MFV-PTC. Like the previously reported case [6], our case was initially misdiagnosed as colloid goiter following both FNA biopsy, as well as thyroidectomy. The peculiarity of our case is that the patient presented a synchronous femoral tumor, which was not previously investigated. It was only after an MRI of the left knee with femur biopsy performed in our institution, that confirmed the presence of bone metastases from a thyroid carcinoma. Histologically, the metastasis was very similar to that observed in the thyroid gland, and which in retrospect was also consistent with a MFV-PTC. Our case is the first in English literature to show femur metastases diagnosed histologically by means of and an image guided core-needle biopsy. Table 1 summarizes some characteristics of the published cases of MFV-PTC metastatic to bone.

**Table 1:** Clinical characteristics of all patients informed with MFV-PTC metastatic to bone

Author (Ref.)	No. of cases	Age/Gender	Average tumor size (cm)	Bones involved	Treatment	Outcome
Cardenas et al. [6]	1	59/W	3.0	Ribs, vertebrae T12, L5 sacrum, righ ischium, and left femoral neck.	RAI 206 mCi of ^131^I	Alive with minimal residual disease.
	2	81/M	12.0	Scapular right.	RAI 206 mCi of ^131^I	Died 6 years later with disease.
Candanedo et al. (Present case) [9]	1	33/M	8.2	Femur diaphysis, epiphysis and sternum handle	RAI 200 mCi of ^131^I and radiotherapy 40 Gy in 10 fractions	Alive 10 months with residual disease

*Ref* References; *W* Women; *M* Man; *RAI* Radioactive Iodine; *Gy* Gray

MFV-PTC represents a source of diagnostic error because it manifest clinically as a thyroid nodule, it can be easily confused with goiter, macrofollicular adenoma, folicular neoplasia or hyperplastic nodule [11], as in our case. Since the 80’s, FNA has emerged as a good diagnostic tool for investigation of thyroid nodules, due to its high diagnostic accuracy and low complication rate [12]. However, FNA biopsy does not seem to be the best investigation to diagnose MFV-PTC. Because, most cases of MFV-PTC are moderately to highly cellular with the presence of microfollicles and macrophages, and the nuclear characteristics of PTC are absent or focal in all cases [13–15]. On the other hand, the image guided core-needle biopsy is a useful method that provides an accurate diagnosis. It allows to determine a possible primary site of origin, using small simples, when we are faced with a metastasis as in our case.

## Conclusion

We have reported the case of a man with MFV-PTC that developed metastasis to the femoral bone. This case confirms the view that MFV-PTC can ocassionally be very aggressive. Only a careful analysis of the architectural and cytological characteristics of goiter or hyperplastic nodules will allow to recognize this rare variety of carcinoma.

## Data Availability

Not applicable. Data sharing is not applicable for this article because no datasets were generated or analyzed during the current study.

## Abbreviations

CT: Computed tomography
^18^F-FDG PET/CT: ^18^F-fluorodeoxyglucose positron emission tomography and computed tomography
FNA: Fine-needle aspiration biopsy
FT3: Free triiodothyronine
FT4: Free tetraiodothyronine
Gy: Gray (a unit derived the dose of ionizing radiation in the International System of Units)
IHC: Immunohistochemistry
MFV-PTC: Macrofollicular variant of papillary thyroid carcinoma
MRI: Magnetic resonance imaging
PTC: Papillary thyroid carcinoma
RAI: Radioactive iodine
TI-RADS: Thyroid imaging reporting and data system
TSH: Thyroid stimulating hormone
TTF-1: Thyroid transcription factor-1
T3T: Total triiodothyronine
T4T: Total tetraiodothyronine
